# Cross Talks between Oxidative Stress, Inflammation and Epigenetics in Diabetic Retinopathy

**DOI:** 10.3390/cells12020300

**Published:** 2023-01-12

**Authors:** Renu A. Kowluru

**Affiliations:** Department of Ophthalmology, Visual and Anatomical Sciences, Wayne State University, Detroit, MI 48201, USA; rkowluru@med.wayne.edu; Tel.: +1-313-993-6714

**Keywords:** diabetic retinopathy, epigenetics, inflammation, oxidative stress

## Abstract

Diabetic retinopathy, one of the most devastating complications of diabetes, is a multifactorial progressing disease with a very complex etiology. Although many metabolic, molecular, functional and structural changes have been identified in the retina and its vasculature, the exact molecular mechanism of its pathogenesis still remains elusive. Sustained high-circulating glucose increases oxidative stress in the retina and also activates the inflammatory cascade. Free radicals increase inflammatory mediators, and inflammation can increase production of free radicals, suggesting a positive loop between them. In addition, diabetes also facilitates many epigenetic modifications that can influence transcription of a gene without changing the DNA sequence. Several genes associated with oxidative stress and inflammation in the pathogenesis of diabetic retinopathy are also influenced by epigenetic modifications. This review discusses cross-talks between oxidative stress, inflammation and epigenetics in diabetic retinopathy. Since epigenetic changes are influenced by external factors such as environment and lifestyle, and they can also be reversed, this opens up possibilities for new strategies to inhibit the development/progression of this sight-threatening disease.

## 1. Introduction

Diabetes is now considered as one of the fastest growing global health emergencies of the 21st century. In 2000, about 150 million people had diabetes, and this number reached to 463 million in 2019. The projections for 2045 are staggering; over 700 million people worldwide are expected to have diabetes. According to the World Health Organization, 6% of the population has diabetes, and 5% are predisposed to diabetes. The disease has a huge burden on the national health system and economy; about one trillion US dollars were spent in 2015, and by 2030, this number is expected to increase to over 2 trillion [1]. Between 2000 and 2019, the mortality rate due to diabetes and its complications increased by over 3%, making it an epidemic of the 21st century.

Diabetes is a life-long disease, and sustained high-circulating glucose, either due to loss of insulin producing beta cells (type 1 diabetes), or the body’s inability to use insulin (type 2 diabetes), affects the entire body, and the damage to the vasculature results in many chronic macrovascular (large vasculature) and microvascular (small vasculature) complications. Some of the major ‘macrovascular’ complications are cerebrovascular and cardiovascular diseases; diabetic patients are over two times more susceptible to heart attack and stroke than nondiabetic individuals [2,3]. The effect of diabetes on the microvasculature is also devastating; end-stage kidney disease is several folds higher in diabetic patients. In fact, diabetes is considered the leading cause of kidney failure. Damage to the nerves is two folds higher in diabetic patients, and diabetes-related issues account for over 50% of nontraumatic amputations [4]. Diabetes-induced damage to the retina (diabetic retinopathy) is the leading cause of blindness in working age-adults. High-circulating glucose is considered the main instigator of diabetic complications, but other systemic factors, including hypertension and hyperlipidemia, also play major roles in their development [5,6]. The molecular mechanisms of the development of these complications, however, remain unclear [7]. Diabetic patients are also at a higher risk of COVID-19 infection and also carry a risk of poor outcomes in COVID-19 infection [8]. Thus, diabetes affects every organ in the body, and also compromises the ability of the immune system to fight infection.

## 2. Diabetic Retinopathy

Diabetic retinopathy affects over 80% of patients after 15–20 years of diabetes (type 1 or type 2). In 2020, 103 million diabetic adults had diabetic retinopathy, and this number included 28.5 million with vision-threatening retinopathy. The projections for 2045 are staggering; 160 million are predicted to have retinopathy, and among those, 44.8 million with vision-threatening retinopathy. Visual impairments, induced by diabetes, negatively impact the patient’s quality of life and their ability to successfully manage their disease; in fact, retinopathy is the most feared complication of diabetes [9]. The two major contributing factors in its development are the duration of diabetes―the longer a person has diabetes, the higher the chances are of developing retinopathy―and the severity of hyperglycemia. In the early stages, the disease remains asymptomatic with no effect on the vision, but as the disease progresses, retinal examination shows progressive retinal damage, first with small bulges in the retinal blood vessels called ‘microaneurysms’. This may be followed by the appearance of cotton wool spots, hemorrhages, lipid deposits and intraretinal microvascular abnormalities. Eventually, this may lead to neovascularization; the new fragile vessels may break and begin to bleed, resulting in vision obstruction. Neovascularization can also lift the retina and detach it, resulting in blindness [10]. Cutting-edge research using experimental models has shown that in the early stages of the disease, the basement membrane thickens and capillary cells undergo accelerated apoptosis, resulting in the degeneration of capillaries and the formation of pericyte ghosts; these are observed before the appearance of any clinical signs of retinopathy [10,11]. Although diabetic retinopathy is considered a microvascular complication of diabetes, nonvascular cells, including retinal neurons and glial cells, also present many metabolic abnormalities and undergo accelerated apoptosis. Furthermore, impaired retinal functions (electroretinograms and contrast sensitivity) are generally seen in diabetic patients before they present any histopathological signs of the disease [12,13].

The etiology of diabetic retinopathy is very complex; circulating hyperglycemia alters many biochemical, molecular and functional pathways. Some of the major pathways affected by hyperglycemia include the formation of advanced glycation end products (AGEs), the polyol pathway and activation of protein kinase C (PKC) and the hexosamine pathway. In addition, diabetes increases reactive oxygen species (ROS), and these pathways produce ROS and are also activated by ROS. Cytosolic ROS damage mitochondria, and mitochondrial damage increases capillary cell apoptosis which ultimately leads to the development of diabetic retinopathy [11,14,15]. Damage to the mitochondria further increases ROS generation, and the vicious cycle of ROS continues to self-propagate. Oxidative stress also increases inflammatory cytokines, and inflammatory cytokines can also increase ROS [16,17,18]. Despite extensive research by leading researchers, the molecular mechanisms of the development of diabetic retinopathy, however, remain elusive, hindering the development of targeted therapeutic interventions.

### 2.1. Oxidative Stress and Diabetic Retinopathy

Free radicals are continuously produced during normal metabolic processes, and intracellular antioxidants neutralize these free radicals so that the body maintains a balance between antioxidants and free radicals [19]. However, many metabolic disturbances shift the balance between free radicals and antioxidants, resulting in excessive free radicals; this could be either due to increased production of free radicals or due to a decrease in the antioxidants, or both. Free radicals are essential for critical redox signaling, but if they are not neutralized, these oxygen-containing molecules with an uneven number of electrons can easily attack proteins, lipids and nucleic acids, leading to cellular dysfunction including altered cell signaling, genetic mutation, energy demand and inflammation [20]. In diabetes, the retina is faced by a double whammy; while free radical production is increased, their neutralization is also impaired due to a compromised antioxidant system [21].

The retina is exposed to both cytosolic ROS and mitochondrial ROS in diabetes. NADPH oxidases (Noxs) catalyze one-electron reduction of oxygen to superoxide anion via oxidizing cytosolic NADPH to NADP [22]. NADPH oxidase 2 (Nox2), a major isoenzyme of the Nox family, is a multiprotein enzyme, and its activation also requires activation of one of the major cytosolic components, Ras-related C3 botulinum toxin substrate 1 (Rac1) [23]. Diabetes activates Nox2 and Rac1, and Rac1-Nox2-ROS signaling is an early event in the pathogenesis of diabetic retinopathy. Increased production of ROS activates matrix metalloproteinases 2&9 (MMP-2 and MMP-9), and with the help of heat shock protein (Hsp60), these MMPs enter the mitochondria. Once inside the mitochondria, they damage the mitochondrial membrane, which activates the apoptosis process by allowing cytochrome c to leak out in the cytosol [24,25].

In normal cellular respiration, ROS are generated during oxidative phosphorylation in the mitochondria by the electron transport chain, and majority of the oxygen entering into the electron transport chain is utilized in the metabolic processes, reducing only 0.1–5% to superoxide [26]. However, in diabetes, increased glucose-derived pyruvate oxidation by the TCA cycle elevates a flux of electron donors into the electron transport chain, increasing the voltage gradient across the mitochondrial membrane; once the critical threshold is reached, electron transfer inside complex III is blocked, resulting in a further increase in superoxide generation [19,27].

In the pathogenesis of diabetic retinopathy, mitochondrial damage-activation of apoptosis follows cytosolic ROS generation, and the damaged mitochondria compromise the electron transport chain system. Mitochondrial DNA (mtDNA) is also damaged, further compromising the electron transport chain. In the initial stages of retinopathy, although mtDNA biogenesis tries to compensate for mitochondrial damage, with time it becomes overwhelmed [28,29], and the damaged mitochondria continue to self-propagate the vicious cycle of ROS [11,30]. Cells are also equipped with very efficient enzymatic and non-enzymatic defense systems, and these systems are damaged in diabetes, creating a double whammy for the retina. Mitochondrial superoxide scavenging enzyme manganese superoxide dismutase (MnSOD, encoded by *Sod2* gene) is inhibited, antioxidant glutathione (GSH) is decreased and the transcriptional activity of the master transcription factor nuclear factor-erythroid 2 related factor 2 (Nrf2)—which regulates antioxidant response elements (AREs)-mediated transcription of antioxidant enzymes—is decreased [25,31]. From the discussion above, it is clear that oxidative stress occupies a major place in the development of diabetic retinopathy.

### 2.2. Inflammation and Diabetic Retinopathy

Diabetes also increases the expression of many inflammatory molecules including cytokines, chemokines and growth factors in the retina. Experimental models have documented the presence of a systemic pro-inflammatory environment with upregulation of CRP, IL-1β, IL-6 and TNFα before the appearance of any pathological characteristic of diabetic retinopathy [32]. The nuclear factor kappa-light-chain-enhancer of activated B, NF-*k*B, a ubiquitous transcription factor important in regulating many cytokines and chemokines, is activated in diabetes [33,34,35], and vitreous and serum of diabetic patients with proliferative retinopathy have elevated levels of many inflammatory mediators including TNF-*α*, IL-1*β*, soluble IL-2 receptor (sIL-2R), IL-8 [36] and several chemokines including CCL2, CCL5, CXCL8, CXCL10. Elevated levels of adhesion molecules such as intracellular adhesion molecule-1 (ICAM-1) and vascular cell adhesion molecule-1 (VCAM-1) are also observed in diabetic retinopathy patients [37]. In addition, NF-*k*B regulates the activation of inflammasomes, and recent studies have shown that the inflammatory cascade is initiated in the glial cells [35], inflammatory cytokines in a hyperglycemic environment activate resident microglia and activated microglia themselves produce inflammatory mediators [38]. Transcriptional changes in activated microglia, via the NF-*k*B signaling pathway, release various pro-inflammatory mediators, including cytokines and chemokines, and the activated microglia also severely affect retinal neurons, thinning the nerve fiber layer [39]. Thus, it is clear that along with oxidative stress, inflammation is also an important factor in the development of diabetic retinopathy.

### 2.3. Oxidative Stress and Inflammation

NF-*k*B is a redox-sensitive transcription factor, and activation of NF-*k*B leads to the initiation of a pro-apoptotic program [33]. As mentioned above, NF-*k*B activation induces proinflammatory mediators, and these mediators in turn further increase ROS production, leading to DNA damage and apoptosis. Furthermore, signaling pathways of the two major transcription factors important for increasing inflammation (NF-*k*B) and oxidative stress (Nrf2) are also shown to cooperate to maintain the physiological homeostasis of cellular redox status and regulate the cellular response to stress and inflammation [40,41]. Nrf2 ameliorates inflammation by inhibiting the transcription of proinflammatory cytokines through direct DNA binding [42], and also negatively controls the NF-*k*B signaling pathway by preventing the proteasomal degradation of I*k*B and inhibiting nuclear translocation of NF-*k*B [43]. Many Nrf2 target genes, such as *Sod2, Rac1* and glutamate-cysteine ligase catalytic subunit (*GCLC*) also have NF-*k*B binding sites, implying their NF-*k*B-mediated regulation [44]. In addition, MMP-9 is a redox-sensitive enzyme, and can be activated via Nrf2 inhibition-NF-*k*B activation [45]. The transcription activity of Nrf2 is reduced, but that of NF-*k*B is increased in diabetic retinopathy, further supporting a close cross-talk between oxidative stress and inflammation [33,46].

MMP-9 is also an important effector molecule in inflammatory cells, and can be involved in both the initial phase of inflammation, by breaking down the pro-cytokines and increasing the cytokine activity [47] and in the later phase of tissue remodeling [48]. MMP activation in diabetes increases vascular permeability in the retina by disrupting the overall tight junction complex and facilitates the tissue availability of the bound vascular endothelial growth factor (VEGF), resulting in neovascularization [49]. Thus, MMP-9, which damages the mitochondria in the early stages, assists in new vessel formation in the development of diabetic retinopathy.

Recent studies have documented a connection between the Nrf2/antioxidant response element (ARE) system and the expression of inflammatory mediators-NF-*k*B pathway [50]. Nrf2, controls the expression of antioxidant genes that ultimately exert anti-inflammatory functions, e.g., Nrf2-regulated heme oxygenase-1 (*HO-1*), which is a potent anti-inflammatory target [51]. Nrf2 also prevents the proteasomal degradation of IkB-α, inhibiting nuclear translocation of NF-*k*B [43]. In addition, the p50 subunit of NF-*k*B can partner with the intracellular inhibitor of Nrf2, Keap1, to form the Keap1-NF-*k*B complex, resulting in the inhibition of Nrf2 transcriptional activity [52]. Nrf2, by competing with the transcription co-activator cAMP response element (CREB) binding protein, can counteract NF-*k*B-driven inflammatory responses [53]. Thus, it is clear that NF-*k*B and Nrf2 have important roles in maintaining both intracellular redox homeostasis and inflammation.

**Figure 1:**
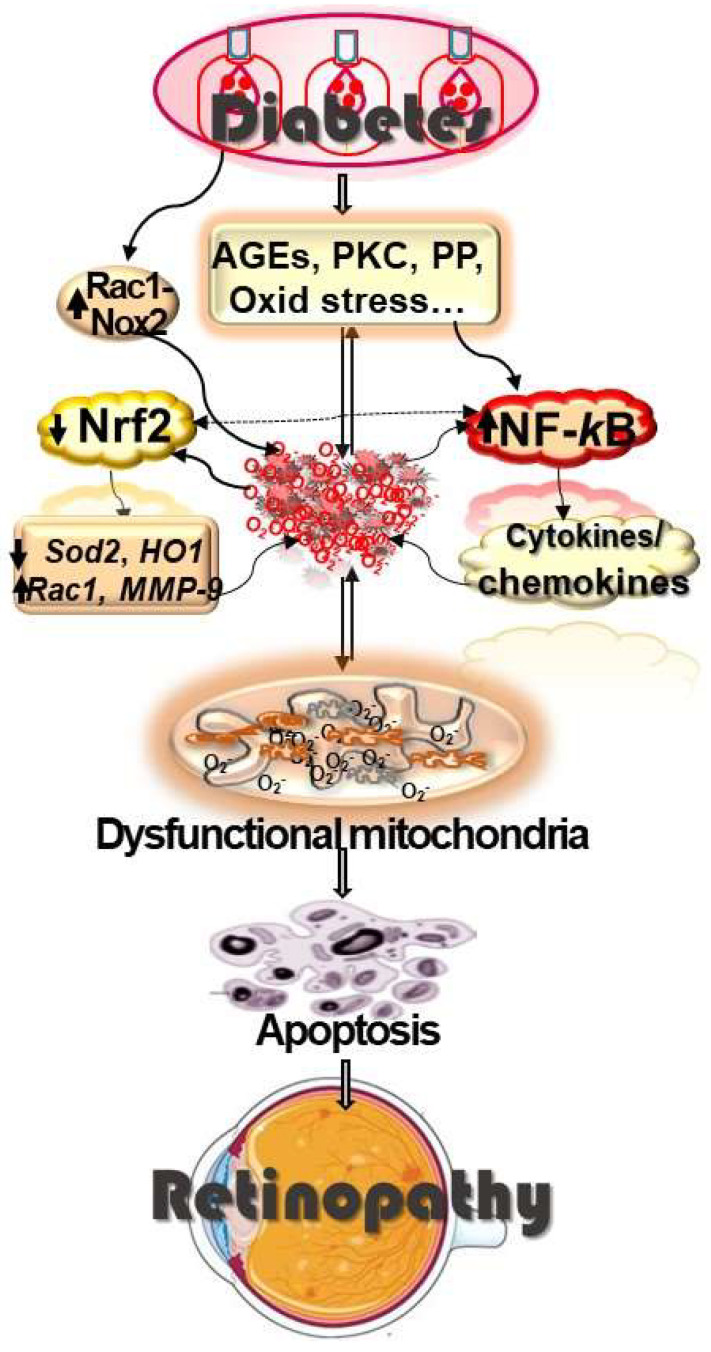
Diabetes alters the activity of many metabolic pathways (AGEs, PKC, polyol pathway, PP), and activation of these metabolic pathways produces ROS; conversely, ROS also activate these pathways. In addition, Rac-Nox2 signaling is activated, which adds to the increase in cytosolic ROS. The transcriptional activity of Nrf2 is decreased, but that of NF-*k*B is increased. Inhibition of Nrf2 transcriptional activity leads to a suboptimal antioxidant defense system, and via NF-*k*B, to the production of inflammatory cytokines, further contributing to increase in oxidative stress and inflammation. Activation of NF-*k*B increases inflammatory cytokines/chemokines, and these inflammatory mediators further fuel ROS production. NF-*k*B activation also transcriptionally activates ROS, producing *Rac1* and *MMP-9* and suppressing ROS-quenching *Sod2*. Cytosolic ROS damage the mitochondria, and the dysfunctional mitochondria accelerate capillary cell apoptosis, resulting in degenerative capillaries and pericyte ghosts. This ultimately leads to the development of diabetic retinopathy.

As mentioned above, the role of nonvascular retinal cells in diabetic retinopathy is gaining recognition; an increased number of microglia, a highly inflammatory related cell type, are seen in diabetic retinopathy, and neuroinflammation is considered to play a significant role in the pathogenesis of diabetic retinopathy [54,55]. Microglia and astrocytes are also two main sources of ROS seen in chronic degenerative neuronal diseases, and in experimental models of diabetic retinopathy, increased production of AGEs and ROS are shown to activate retinal microglia, increasing the production of cytokines, TNF-α and IL-β [56]. In addition, increased oxidative stress damages retinal endothelial cells, increasing the permeability of the microvascular cells, and this facilitates recruitment of inflammatory cells [57]. Thus, it is clear that there is a close cross-talk between oxidative stress and inflammation in the pathogenesis of diabetic retinopathy (Figure 1).

## 3. Epigenetics

Recent technological advancements have documented that genes can be turned on (overexpress) or off (repress) without altering the DNA sequence. These epigenetic changes, mainly DNA methylation, histone posttranslational modifications and non-coding RNAs, regulate gene expression and are associated with both normal cellular differentiation and development of diseases. Epigenetic modifications are rapid; they can be reversible or partly transmissible to the next generation and are facilitated by a dedicated group of enzymes. The “writers” bring in a group, the “readers” read and recognize them and the “erasers” remove them [58,59].

### 3.1. DNA Methylation

DNA methylation, one of the major epigenetic modifications, is generally considered a gene repressing modification. Transfer of a methyl group onto the C5 position of the cytosine, facilitated by DNA methyl transferases (Dnmts), forms 5-methylcytosine (5mC), which condenses the chromatin and impedes the binding of transcription factors. In normal physiological conditions, the CpG islands at gene promoter regions are generally unmethylated and are susceptible to methylation by external factors and disease states. Methylated cytosine, however, is also prone to spontaneous deamination to thymine, and the rate of 5mC to thymine formation is much higher than that of unmethylated cytosine [60,61,62]. Methylation of DNA is a very dynamic process, and with the help of active ten-eleven translocases (Tets), 5mC can be rapidly oxidized to 5-hydroxymethyl (5hmC), which relaxes the chromatin, resulting in gene activation [63,64]. Thus, although DNA methylation results in gene suppression via hydroxymethylation of methyl cytosine, it can also activate gene expression.

### 3.2. Histone Modifications

Nucleosomes have highly orchestrated DNA packaging formed by the repeating units of the tetrameric structure consisting of histones 2 (H2A &B), H3 and H4; however, the N-terminal sequences of the histones is available for modifications, including acetylation and methylation. These posttranslational modifications alter the chromatin structure and regulate the gene expression by altering the binding of transcription factors to their *cis*-regulatory elements. Histone acetylation generally activates gene expression, and is maintained by a balance between histone acetyltransferases (HATs) and histone deacetylases (HDACs). Aberrant histone acetylation/deacetylation is implicated in many pathologic conditions, including inflammatory and degenerative diseases [65,66]. Another major histone modification is histone methylation. Histone methylase adds a methyl group on the lysine tail and histone demethylase removes a methyl group. Unlike acetylation, histone methylation can either suppress or activate gene expression, and this depends on the site of methylation and the number of methyl groups. For example, dimethylation (H3K4me2) can result in both inactive and active euchromatic genes; trimethylation of lysine 4 on histone H3 (H3K4me3) is associated with an active gene expression and dimethylation at lysine 9 (H3K9me2) in gene silencing [67].

### 3.3. Noncoding RNAs

Technological advancements in transcriptome-wide sequencing have revealed that only 2–3% of mammalian genes are transcribed into RNAs that translate into protein, and over 80% of the mammalian genome consists of noncoding RNAs, RNAs without any open reading frame for translation [68]. These noncoding RNAs can be micro RNA (miRNA, less than 20 base pairs), long noncoding RNA (LncRNAs, more than 200 base pairs), transfer RNA and circular RNA. Noncoding RNAs are capable of attaching to the coding region of a gene or recruiting proteins to modify the histone and alter expression of a gene [58,69,70]. The human genome project has documented that many of the noncoding RNAs can act as epigenetic components to regulate gene expression. For example, miRNAs, a class of single-stranded RNA molecules, can influence the output of protein-coding genes by targeting mRNAs for cleavage or translational repression [71,72], and LncRNAs, by remodeling chromatin and genomic imprinting or sequence complementarity with RNAs or DNAs, can regulate gene expression [73]. Although the miRNA field has received a great deal of attention and ~2000 miRNAs have been identified, despite the large numbers of LncRNAs identified thus far (~270,000) [74], the field is still in its early stages.

**Figure 2:**
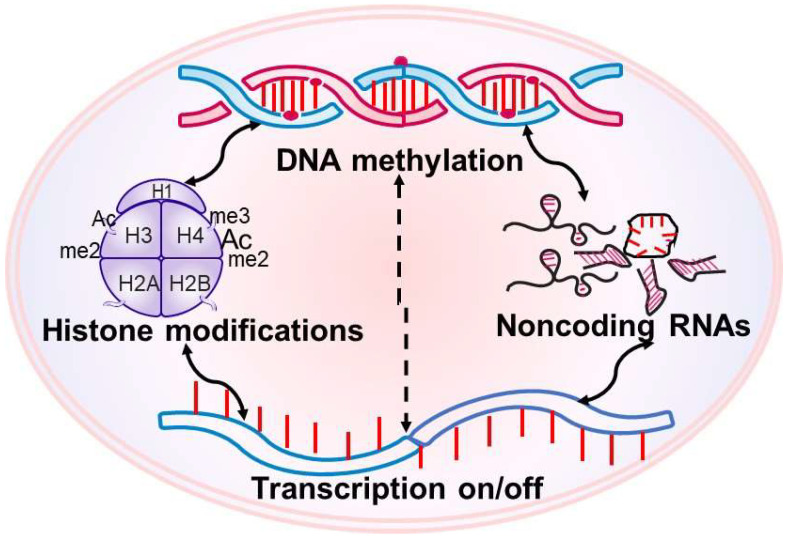
Epigenetic modifications are interrelated; DNA methylation can regulate histone modifying enzymes and can also regulate noncoding RNAs. Noncoding RNAs can regulate the machinery essential for DNA methylation and histone modifications.

Epigenetic modifications also cross-regulate each other, diversifying their functional states, and the cross talk can originate from one modification (e.g., histone methylation) altering the activity of the enzyme responsible for other modification (e.g., Dnmts). Moreover, over 50% of miRNA genes are associated with CpG islands, and miRNA can directly target and suppress the mRNAs of Dnmts [75]. LncRNAs can mediate DNA methylation in the promoter regions and can also be regulated by DNA methylation and interact with miRNAs [76] (Figure 2).

## 4. Epigenetics, Oxidative Stress and Diabetic Retinopathy

The role of epigenetics in diabetic retinopathy is still in very early stages; analysis of family history in a cross-sectional study of type 2 diabetes has shown some genetic and epigenetic associations with the development of diabetic retinopathy [77,78]. Case studies have reported significantly higher levels of global DNA methylation in type 2 diabetic patients with retinopathy compared to those with no retinopathy, and have reported a correlation between the methylation status of DNA and the progression of retinopathy [77,78]. Additionally, over two hundred unique genes that are associated with proliferative diabetic retinopathy have shown differential DNA methylation [79], and higher global DNA methylation is reported in the leukocytes from type 2 diabetic patients with retinopathy compared to those with no retinopathy [77]. Our study has shown an increase in methylation of mtDNA in the peripheral blood of diabetic patients with proliferative diabetic retinopathy compared to patients without retinopathy [80].

As mentioned above, experimental models have revealed that generation of cytosolic ROS via Rac1-Nox2 signaling and the activation of MMP-9 are early events in the pathogenesis of diabetic retinopathy [23,24,25]. The promoters of *Rac1* and *MMP9* undergo dynamic DNA methylation-hydroxymethylation; while increased binding of Dnmt1 at their promoters hypermethylates the DNA, concomitant increased binding of Tets rapidly converts 5mC to 5hmC, resulting in increased expressions of *Rac1* and *MMP-9*. In addition, mtDNA itself is also hypermethylated, and its transcription is impaired. To make this bad situation worse, DNA at the promoter of DNA mismatch repair enzyme MutLH1 (*MLH1*) is also hypermethylated, and its expression is suppressed, further contributing to the mtDNA damage. Hypermethylation of the promoter DNA of polymerase gamma (*POLG*), critical for mtDNA biogenesis, decreases mtDNA copy numbers [11,25,81]. As mentioned earlier, mitochondria are very dynamic, and a balance between their fusion and fission is critical for responding to the varying energy demands and stability. DNA methylation at the promoter of mitochondrial fusion protein mitofusin 2 (*Mfn2*) decreases its expression, and DNA methylation-hydroxymethylation of the promoter of *Drp1*, a mitochondrial fission protein, increases its expression [82]. Thus, in addition to DNA methylation playing a major role in mitochondrial damage (hydroxymethylation of *Rac1* and *MMP-9* and hypermethylation of mtDNA and Mlh1), hypermethylation of *POLG* reduces mitochondrial copy numbers, and by altering the DNA methylation status of *Mfn2-Drp1*, it also impairs mitochondrial dynamics. Furthermore, altered histone modifications at the promoter of *Sod2* reduces its expression and its scavenging capacity to remove mitochondrial superoxide [83,84]. Altered H3K4 methylation at Keap1 in diabetes is implicated in its gene activation, resulting in restraining Nrf2 in the cytosol; this impairs Nrf2 transcriptional activity and via *GCLC*-ARE4 reduces GSH biosynthesis [51,85]. 

Several noncoding RNAs, including miRNAs and long noncoding RNAs, are also implicated with oxidative stress in diabetes. MicroRNAs miR-200c and miR-383 promote ROS production in diabetes, and miR-145 and miR-383 inhibit oxidative stress and retinal endothelial cell apoptosis [86]. Furthermore, miR-7, 15a, 27b, 100, 195, 200b, 365 and miR-455–5p are also associated with oxidative stress [87]. Upregulation of Lnc*ANRIL*, Lnc*MALAT1* and Lnc*NEAT1* increases ROS and mitochondrial damage [88,89], and our recent study has documented that via affecting the interactions of Nrf2 and its intracellular inhibitor Keap1, Lnc*MALAT1* upregulation suppresses the transcriptional activity of Nrf2, thus impairing the transcription of antioxidant defense genes such as *HO-1* and *Sod2* [88]. Thus, in the pathogenesis of diabetic retinopathy, epigenetic modifications including DNA methylation, histone modifications and noncoding RNAs modulate oxidative stress-mitochondrial damage.

## 5. Epigenetics, Inflammation and Diabetic Retinopathy

The role of inflammation in diabetic retinopathy is now being implicated by several leading labs, but knowledge of the role of epigenetics in the regulation of the inflammatory cascade in diabetic retinopathy is still in its incipient stages. DNA hypomethylation of NLR family pyrin domain containing 3 (*NLRP3*) is associated with increased risk of diabetic retinopathy [90], and that of *TNFα, TGFβ1* and *MCP-1* is considered responsible for its increased expression in the serum of patients with proliferative diabetic retinopathy [79]. Experimental models of diabetic retinopathy have shown that silencing thioredoxin interacting protein *TXNIP* prevents epigenetic modifications in the promoter of cyclooxygenase 2 (*Cox2*), an enzyme important in the synthesis of a major mediator of inflammation, prostaglandin E2 (*PGE2*) [91].

Many miRNAs are also associated with the regulation of inflammation in diabetic retinopathy, e.g., downregulation of miR-146a, miR-15a/16 and miR-20b-3p is associated with increased proinflammatory cytokines and leukostasis [92,93], and downregulation of miR-15a with pro-inflammatory and pro-angiogenic changes [94]. MiR-204 inhibits inflammation and cell apoptosis in rats with diabetic retinopathy via upregulation of sirtuin 1 [95]. In addition to miRNAs, recent research has associated many LncRNAs with inflammation in diabetic retinopathy; e.g., Lnc*MALAT1* is considered an epigenetic regulator of inflammation in diabetic retinopathy [96], and Lnc*ANRIL* is implicated in increased VEGF [97]. Another LncRNA, *HOX* antisense intergenic RNA (*HOTAIR*), is upregulated in the vitreous of diabetic retinopathy patients and in retinal endothelial cells incubated in high glucose, and its inhibition is shown to prevent an increase in retinal vascular permeability and VEGF in diabetic rodents [98].

## 6. Cross-Talks between Epigenetics, Oxidative Stress and Inflammation

As stated above, activation of the redox sensitive NF-*k*B leads to induction of proinflammatory mediators, and these mediators, in turn, can produce ROS [99]. NF-*k*B-mediated signaling is negatively controlled by the master regulator Nrf2 [40,41]. DNA methylation-hydroxymethylation affects the binding of the p65 subunit of the transcription factor NF-*k*B at the promoters of *Rac1* and *MMP-9*, transcriptionally activating their expressions and increasing oxidative stress [100,101]. Upregulation of poly(ADP-ribose) polymerase-1 (PARP-1), a regulator of transcription factor binding on a gene promoter, in diabetes, is implicated in the increased binding of NF-*k*B at *MMP-9* promoter, increasing its transcription [102]. Epigenetic modifications of the antioxidant machinery also affect the binding of NF-*k*B; for example, histone modifications at the promoter and enhancer of *Sod2* in diabetes alter the binding of the p65 subunit of NF-*k*B and suppress its transcription [83]. Histone deacetylase, which has specific affinity towards *ARE*-sequences, inhibits the binding of the p65 subunit of NF-*k*B to the gene promoter region and suppresses the Nrf2-*ARE* signaling pathway, suggesting the direct involvement of histone deacetylase in the regulation of Nrf2-dependent antioxidant signaling [103]. Moreover, histone modification at the promoter of *GCLC-ARE4* is implicated in its gene suppression-subnormal levels of the intracellular antioxidant GSH [51].

Many miRNAs that regulate both oxidative stress and inflammation have now been identified; for example, via increasing oxidative stress, upregulation of miR-365 is associated with Müller cell gliosis [104], and miR-145 attenuates high glucose-induced oxidative stress and inflammation in retinal endothelial cells through regulating TLR4/ NF-*k*B signaling [105]. Intravitreal administration of miR-146 inhibits diabetes-induced - NF-*k*B activation and retinal microvascular and neuronal functional defects [106]. 

In addition to miRNAs, Lnc*MALAT1* expression is augmented by hypoxia, contributing to the proliferative response in endothelial cells [107], and inhibition of Lnc*MALAT1* in retinal endothelial cells ameliorates cell migration-angiogenesis, neurodegeneration and MCP-1 [108,109]. Furthermore, experimental models of diabetic retinopathy have shown that Lnc*MALAT1*, via associating with polycomb repressive complex 2 (PRC2) components, regulates the expression of several inflammatory genes [96], and LncRNA *H19*, via the TGF-β-mediated pathway, regulates endothelial-mesenchymal transition [110]. Lnc*HOTAIR* mediates angiogenesis via epigenetically activating VEGF-A [98] and Lnc*ANRIL* tube formation-proliferation in endothelial cells, via regulation of VEGF expression [97]. Another LncRNA, myocardial infarction associated transcript (Lnc*MIAT*), is regulated by NF-*k*B; high glucose increases the NF-*k*B and *MIAT* binding, further regulating inflammatory cytokines and apoptosis [111]. LncRNA maternally expressed gene 3 (Lnc*MEG3*), which increases endoplasmic reticulum stress and inhibits cell proliferation [112], is considered to negatively correlate with VEGF, and in patients with diabetic retinopathy, while the serum levels of VEGF are upregulated, Lnc*MEG3* are downregulated [113]. Thus, the role of epigenetics, including noncoding RNAs, inflammation, oxidative stress and angiogenesis, in the pathogenesis of diabetic retinopathy, is now gaining recognition.

## 7. Conclusions

In conclusion, advances in technology have facilitated the diagnosis of diabetic retinopathy and have helped in solidifying the role of oxidative stress, inflammation and epigenetics in its development. However, the multifactorial nature of this slow progressing disease has made it difficult to identify specific therapeutic modalities to treat this blinding disease that a diabetic patient fears the most. [114]. Additionally, pan-retinal photocoagulation, though highly effective for treating proliferative diabetic retinopathy, can cause unwanted effects, including choroidal effusions, exudative retinal detachments and visual field deficits [115]. As in the discussion presented above, epigenetics modifications play a major role in both oxidative stress and inflammatory cascades associated with the development of diabetic retinopathy, and these modifications are influenced by external factors including lifestyle and environment, changing how a DNA sequence is read and genes are expressed. Fortunately, unlike genetic mutations, epigenetic changes can also be reversed by changing one’s lifestyle and by novel therapeutics that are currently in the pipeline, making the future of inhibiting the development or progression of retinopathy optimistic for diabetic patients.
